# Archetypal typology of European forest ecosystems integrating management intensity and naturalness

**DOI:** 10.1007/s13280-024-02050-3

**Published:** 2024-07-11

**Authors:** José I. Barredo, Matteo Vizzarri, Klára Kuželová

**Affiliations:** 1 European Commission, Joint Research Centre (JRC), Via Enrico Fermi, 2749, 21027 Ispra, VA Italy; 2 Università Degli Studi Di Milano, Via Festa del Perdono 7, 20122 Milan, Italy; 3 ARHS Developments S.A., Rue Nicolas Bové, 2B, 1253 Luxembourg, Luxembourg

**Keywords:** Archetype, Ecosystem, Europe, Forest, Management, Naturalness

## Abstract

The crises of climate change and biodiversity loss have pushed the aim for increasing the resilience of forest ecosystems high on the agenda of foresters and policymakers. At the same time, synergistic opportunities for restoring forests and biodiversity are emerging to safeguard these ecosystems. Naturalness is a key characteristic of forest ecosystems, which should be considered when estimating benchmarks for resilience and biodiversity conservation. The naturalness of forest ecosystems is highly dependent on the intensity of human activity, as different levels of management intensity can change the original traits of forest ecosystems. This paper presents an archetypal typology of forest ecosystems, describing the association between management and naturalness. Both features are represented as gradients covering the full spectrum observed in European forests. The array of forest ecosystem archetypes was verified using case studies across Europe. The typology provides useful information for setting targets for resilience and restoration of forest ecosystems.

**Supplementary Information:**

The online version contains supplementary material available at 10.1007/s13280-024-02050-3.

## Introduction

Forest ecosystems are subject to the interlinked global crises of climate change and biodiversity loss (Pörtner et al. 2021; Mahecha et al. 2022). Moreover, these ecosystems in Europe are affected by changes in disturbances regimes posing new challenges (Trumbore et al. 2015; Seidl et al. 2017; McDowell et al. 2020; Senf and Seidl 2021). Consequently, enhancing the resilience of forest ecosystems has become a priority for forest managers and conservation policy (European Commission 2023; Lindner et al. 2023). It is not a coincidence that the term 'resilience' is mentioned in 22 instances across the 27 pages of EU Forest Strategy to 2030 (European Commission 2021). Achieving such an aim in managed forests requires management approaches oriented towards mimicking features of natural forests, which are beneficial in halting the loss of forest biodiversity and contribute to ecosystem restoration efforts. In other words, this involves creating more diverse forests with varied age structures, tree species, genetic diversity, and other taxa diversity, as well as increased horizontal and vertical structural complexity (Thompson et al. 2009; Lindner et al. 2023).

Naturalness is a characteristic of forest ecosystems resulting from the historical evolution of forests, land use legacies, and current forest management (Poeplau and Don 2013; McGrath et al. 2015; Munteanu et al. 2015; Felipe-Lucia et al. 2018; Chiarucci and Piovesan 2020; Mayer et al. 2020). The degree of naturalness in forest ecosystems largely depends on the time since the last silvicultural intervention and the intensity of management, which shapes forest traits based on management objectives (Korjus and Laarmann 2015). Different degrees of management intensity result from the level of human manipulation of the processes of forest development. That is, deliberate alterations of forest traits oriented to enhanced wood production (Duncker et al. 2012a). The degree of naturalness reflects the transition from natural complex ecosystems to simplified forests aimed at wood production. This is because, depending on the management objective, forest management can directly influence various forest traits. These include tree species composition and diversity, structural diversity (such as canopy layers and age structure), amounts of lying and standing deadwood, the type of regeneration, canopy closure, development phases, and the diversity of epiphytic lichens, mosses, and herb layer species, (Winter et al. 2010; Winter 2012; Korjus and Laarmann 2015; Chiarucci and Piovesan 2020; Meyer et al. 2021; Edelmann et al. 2022). Forest management can also affect other forest characteristics, such as nutrient levels and the amount of soil organic carbon, through indirect pathways (Penuelas and Baldocchi 2019).

The adoption of forest management approaches aimed at increasing the resilience of forest ecosystems necessitates an understanding of how such management affects their naturalness. Resilience is defined as the capacity of an ecosystem to return to its original state following a perturbation while maintaining its characteristic taxonomic composition, structures, functions, and process rates (Holling 1973; Thompson et al. 2009). Put simply, to define resilience targets, we need to know about the relationship between the varying levels of management intensity and the resulting degree of naturalness. Furthermore, this information can elucidate the potential impacts of changes in management intensity on the naturalness of forests. Consequently, it is essential to describe interdisciplinary linkages to understand how societal drivers affect ecosystems, the feedbacks that result from societal changes, and the projected outcomes of decision-making processes (Clark et al. 2001). This entails generalisations and predictions about how ecosystems will respond to changes in management intensity. Although there is abundant scientific literature on both forest naturalness and management, a comprehensive framework that integrates the full spectrum of management intensity and degrees of naturalness for European forests is currently lacking. The aim of this paper, therefore, is to close this gap. It seeks to do so by delineating the relationship between management intensity and naturalness and by introducing a conceptual archetype typology of forest ecosystems, which integrates explicitly both forest management intensity and naturalness. The focus is specifically on European forest ecosystems. This typology is expected to be a useful resource for forest managers, conservation biologists, and policymakers, assisting them in formulating strategies to bolster resilience and preserve biodiversity within these crucial ecosystems.

## Conceptual framework

### Forest naturalness

Forest naturalness is defined as ‘the similarity of a current ecosystem state to its natural state’ (Winter et al. 2010; Winter 2012); naturalness varies according to the degree of anthropogenic influence on the ecosystem (Sukopp et al. 1990; Angermeier 2000; Machado 2004; McRoberts et al. 2012; Keith et al. 2020). It is a characteristic of forest ecosystems resulting from human influence affecting functions, composition, and structure (Roberge et al. 2008; McRoberts et al. 2012; Winter 2012). Forest naturalness is often described as a gradient from ‘intact’ primeval forest ecosystems to simplified tree dominated areas such as forest plantations (Buchwald 2005; Roberge et al. 2008; McRoberts et al. 2012; Winter et al. 2013). This creates a gradient that is often associated with decreasing levels of forest ecosystem services and biodiversity, from the most natural forests possible under current conditions to the more human-modified forests of the gradient (Winter et al. 2010; Duncker et al. 2012b; Winter 2012; Gamfeldt et al. 2013; Pukkala 2016; Sing et al. 2017; Smith et al. 2017; Santos-Martín et al. 2019; Qiu et al. 2021; Kärvemo et al. 2023).

The concept of naturalness has a broad meaning that includes various anthropogenic effects, such as forest fragmentation or the effects of pollutants. However, in this study, we limit the notion of naturalness to the effects derived solely from forest management at stand level. In line with this perspective, several assessments of forest naturalness have focused exclusively on forest traits influenced by management practices (Liira and Sepp 2009; McRoberts et al. 2012; Korjus and Laarmann 2015).

Buchwald (2005) proposed a hierarchical gradient of forest naturalness created using information on the following forest features: origin and genesis of the stand, origin (natives, exotic) and provenance (breeding facilities, nurseries, wild environment) of tree species, forest processes and structures, forest continuity, management objectives, and forestry activities. The gradient consists of 14 mutually exclusive degrees of forest naturalness (Table 1). In Europe, the first three categories (n10 to n8) are confined to some patches in Northern Fennoscandia and areas of European Russia (Sabatini et al. 2018, 2020). Therefore, in this study, we considered these categories as being covered by category n7: Near virgin forests. Buchwald (2005) described each naturalness level including features such as forest management, information on the origin and genesis of the stands, structural features, and tree species composition. A more detailed description of the naturalness categories is in Table S1.

**Table 1:** Levels of forest naturalness, short description of the levels, and spatial scale. Each level of naturalness is associated with a series of ecological characteristics and condition features, which provide a preliminary understanding of the intensity of forest use across different levels of naturalness. The spatial scale of each naturalness level is indicated in two categories, i.e. landscape and stand. Categories n8 to n10 not shown because they are very marginal in Europe, these categories were considered to be covered by category n7. Source: modified from Buchwald (2005)

Level of forest naturalness	Short description	Spatial scale
n7–Near-virgin forest	Structures, dynamics, and species composition similar to primary forests, even though they may have been modified by human action in the past	Landscape, stand
n6–Old-growth forest	Characterised by old trees and related structural attributes. Encompasses the later stages of stand development. Other characteristics are large trees for species and site, wide variation in tree sizes and spacing, accumulations of large amounts standing and lying deadwood, multiple canopy layers. This level may show signs of past human disturbance, but these are limited so as not to disrupt natural processes	Stand
n5–Long-untouched forest	Relatively intact forest unmodified by human activity for the past sixty to eighty years or for a relatively long time. Signs of former human impacts may still be visible, but strongly blurred due to the decades without forestry operations	Stand
n4–Newly untouched forest	Forestry operations have been discontinued or never occurred since stand establishment, and which have been left untouched for less than sixty to eighty years. If the suspension of management operations is solely due to long intervals between forestry operations, the stand should be classified at lower levels	Stand
n3–Specially managed forest	Low-intensity use and presence of old-growth attributes. Significant biodiversity value. Examples are coppice, pasture forests, non-industrial selective logging, and stands of low accessibility or with protective or recreational functions	Stand
n2–Exploited natural forest	Managed forest so that the forest structure and species composition is significantly changed from the originally natural state, but still predominantly consisting of self-sown native trees, and without a plantation-like structure	Stand
n1–Plantation-like natural forest	Predominantly consisting of self-sown native trees with high-intensity forest management. Forest structure is plantation-like by being even-aged, having relatively low tree ages, fairly regular tree spacing, and only one or two tree species in the canopy layer	Stand
p4–Partly natural planted forest	Predominantly consisting of native trees that are planted or sown, these forests have an uneven-aged structure, mixed species, and significant ingrowth of self-sown trees	Stand
p3–Native plantation	Even-aged forests predominantly consisting of native trees established artificially by planting or sowing with regular spacing. Often monospecific stands, but occasionally two or more species are established together	Stand
p2–Exotic plantation	Predominantly consisting of even-aged non-native tree species where stand origin is artificial by planting or sowing	Stand
p1–Self-sown exotic forest	Predominantly consisting of self-sown non-native tree species. Forests in this category can spread at an undesirable scale to the extent that it has replaced or seriously suppressed native species previously occupying the area	Stand

The categories of the naturalness gradient are described using two spatial units, namely the stand and landscape level. A forest stand is a contiguous community of trees, uniform in terms of composition, structure, age, size, class distribution, spatial arrangement, or location on a site of uniform condition (Nyland 2007). In turn, the landscape scale describes arrays of heterogeneous forest stands that form the forest landscape mosaic (Seymour and Hunter 1999). Operational studies of forest naturalness are often implemented using the stand as the minimum spatial unit of assessment (Çolak et al. 2003; Bartha et al. 2006; McElhinny et al. 2006; Liira and Sepp 2009).

### Forest management

Forest management is defined as ‘the process of planning and implementing practices for the stewardship and use of forests to meet specific environmental, economic, social and cultural objectives’ (FAO 2020). Consequently, management objectives drive decisions about management practices. For instance, decisions may be made to prioritise one forest service over others, or conversely, to target the supply of the full range of forest services. These include, among others, wood, fresh water, erosion protection, food, recreational options, and carbon storage and sequestration. Therefore, the management objective is central to the adoption of one forest management approach over others, delineating the range of silvicultural operations and decisions adopted by forest managers.

In the European context, Duncker et al. (2012a) proposed a gradient of five forest management approaches based on management intensity, i.e. from unmanaged or conservation management to intensive management (Table 2). The adoption of one management approach influences the development of forests, thus affecting structural, functional and compositional traits, and consequently the supply of forest ecosystem services (Duncker et al. 2012b). Although silvicultural practices can be combined in several ways, the framework of Duncker et al. (2012a) provides a comprehensive view of the main forest management approaches existing in Europe and its associated silvicultural practices. However, these approaches are not mutually exclusive, as the range of options allows for greater flexibility in selecting silvicultural operations.

**Table 2:** Forest management approaches, forest use intensity and short description. Source: modified from Duncker et al. (2012a), and from European Commission (2023) for closer-to-nature forestry

Forest management approach	Intensity	Short description
Unmanaged or conservation forests	Passive	Natural processes and natural disturbance regimes can develop without direct human disturbances. Conservation goals are given primacy
Closer-to-nature forestry	Low	The aim is to manage stands by emulating natural processes. Economic return is important but must occur within this aim. Silvicultural disturbances should resemble the natural disturbance regime in terms of spatio-temporal patterns and the amount of trees removed, allowing for natural regeneration. Management interventions must enhance or conserve the ecological functions of the forest. For instance, standing and fallen deadwood must remain in the forest
Combined objective forestry	Medium	Various management objectives are combined in a manner that meets a compromise among the provision of different ecosystem services within a common management approach. Both economic and ecological aims play major roles. This involves timber production, but is also associated with habitat, water, soil protection, and nature conservation. Native or introduced tree species suitable for the site can be used. Natural regeneration is the preferred method, but planting or seeding is acceptable. Tree species mixtures are typical for the forest type
Intensive even-aged forestry	High	Characterised by stands in which no, or relatively small, age differences occur among individual trees. In this approach, stands are even-aged with only one (occasionally two, if that increases or diversifies wood production) tree species, with the objective being timber production
Short rotation forestry	Intensive	The main objective is to produce the highest amount of merchantable timber or wood biomass. Economic objectives are prioritised over ecological concerns

Forest management approaches are defined by sets of forest operations at the stand level. For this reason, the stand is generally the spatial unit of reference for operational applications. A more detailed description of the management categories is in Table S2.

### Legacies of earlier activity

Understanding the historical evolution of forests is crucial in assessing the naturalness of forest ecosystems. In this context, we offer a summary of the history of European forests that aids in comprehending their current degree of naturalness. After the Last Glacial Maximum ended 27 000–19 000 years ago, the European continent was covered by natural landscapes (Kaplan et al. 2009; Tallavaara et al. 2015). Yet, over the last six millennia, European forests have undergone extensive modifications due to intensive use, management, and anthropogenic disturbances (Kaplan et al. 2009; Schulze et al. 2009). In particular, deforestation for conversion of forests to agricultural and pasture land, and forest clearing for wood use was prevalent from 1000 BC until the first half of the last century. Reconstructions of the evolution of forests in Europe indicate major forest clearance between 1000 BC and AD 1850 (Kaplan et al. 2009). By the period around 1000 BC, most of the European continent retained its forest cover. However, in the period between AD 350 and AD 1000, net forest clearance remained the general trend in Central and Western Europe, with many regions reaching deforestation levels of 80% to 90%. The highest levels of deforestation were reached between AD 1500 and AD 1850, including Eastern Europe for the first time history. In Fennoscandian boreal forests, the effects of human activity began in the early 1800s with the onset of preindustrial forest utilisation. However, it was the industrial forest exploitation starting around 1860 that introduced major changes to Fennoscandian forests. This was followed by intensive forest management from around 1920 to the present, leading to significant changes in forest naturalness and losses in biodiversity (Östlund et al. 1997). Deforestation was estimated at between 60 and 100% across European regions by AD 1800–1850, considered a low point in forest cover (Kaplan et al. 2009; McGrath et al. 2015). Forest degradation reached its maximum in this period due to forest over-use for firewood, the production of charcoal, and supplies for continuing wars (Schulze et al. 2009). In addition, there was a shift initiated about AD 1700 from broad-leaved forests to more productive conifers. McGrath et al. (2015) estimated an increase of 593 000 km^2^ of coniferous forests at the expenses of deciduous forests, decreasing by 538 000 km^2^, an area equivalent to around one-third of the current extent of forest in the EU, between 1600 and 2010.

The legacies of the devastation of forests in Europe may still be visible today. For example, in the decreased amount of soil carbon and nutrients in forest areas where land use changed from forest to agriculture, and then from agriculture returning to forest again (Kaplan et al. 2009; Poeplau and Don 2013; Mayer et al. 2020). In contrast, the long-term legacies of forest use for pasture, such as wooded meadows and wooded pastures, which were significant in Europe, are also considered to play a role in today’s ecosystems due to their potential biodiversity benefits (Kull and Zobel 1991; Thor et al. 2010; Hejcman et al. 2013). Land use and land cover reconstructions of the past century indicate that after the timber shortage of the Second World War, reforestation and afforestation actions were significant across much of the European continent, including mountainous regions (Fuchs et al. 2015; Munteanu et al. 2015). At present, it is estimated that primary and old-growth forests account for less than 3% of Europe’s forests (Sabatini et al. 2020; Barredo et al. 2021), and 77% of the forest area and 84% of the growing stock of European forests are available for wood supply (FOREST EUROPE 2020). This means that most European forests fall into potentially production-oriented forest land. In addition, more than 70% of European forests are even-aged (FOREST EUROPE 2020), which indicates forest ecosystems with structural features far from a natural condition. This is corroborated by the study conducted by Strona et al. (2016), which examined the structure of tree assemblages in European forests.

## Materials and methods

The archetype typology was developed by evaluating the similarities between the traits of each of the 11 categories of forest naturalness, as described by Buchwald (2005), and the traits of the 5 categories of management intensity, as outlined by Duncker et al. (2012a). This association yields 55 (11 × 5) possible combinations. The similarities between the categories of naturalness and management intensity were calculated in four steps. First, we compiled a list of 19 forest indicators commonly used to describe the naturalness and management characteristics of forests (Buchwald 2005; Winter et al. 2010; Duncker et al. 2012a; McRoberts et al. 2012). We organised these indicators into four categories: structural, functional, compositional, and human impact. Subsequently, we assigned a set of descriptors to each indicator (Table S3).

Second, for each category of naturalness and management, we assigned at least one descriptor to each indicator, as shown in Table S3. We based these assignments on information from Buchwald (2005) and Duncker et al. (2012a), which describe the frameworks adopted for naturalness and management, respectively. Consequently, we generated a 19-component vector for each category of naturalness and management, where each component corresponds to an indicator. Some components contain multiple descriptors when an indicator is associated with more than one descriptor within a given category.

Third, we calculated the Hamming distance between the vectors representing the naturalness and management categories to measure their degree of similarity (Hamming 1950, 1980; Klenk et al. 2008; Sirovich et al. 2010). The Hamming distance is a metric that quantifies the proportion of differing components in two vectors of the same dimension. Specifically, a distance of 0 (zero) indicates that the vectors are identical, whereas a distance of 1 indicates that they are completely different. For instance, the Hamming distance between vector A [1, 2, 1, 0, 3] and vector B [1, 2, 2, 0, 3] is 0.2. That is, one out of five, or 0.2, components did not match. For components with multiple descriptors, we used the 'or' logical operator, which returns 'true' if at least one descriptor matches the component being compared. For example, the components [2, 3] and [3] would be considered to match.

Fourth, we populated a matrix with the Hamming distance calculated for each of the 55 combinations of naturalness and management categories. We then used this matrix to delineate the archetypes on a per-row basis. Specifically, for each row representing a category of naturalness, we identified the archetype by selecting cells that (1) exhibited the lower Hamming distances and (2) demonstrated a plausible association with the corresponding management category, as shown in Table S4. This second step utilised ancillary information from Tables S1 and S2. Forests in the naturalness categories n5, n6, and n7 are defined as primary forests by Buchwald (2005); therefore, they were aggregated into one archetype.

We verified the representativeness of the resulting archetypes by using information from case studies describing different types of forest ecosystems in Europe. Information on case studies was collected in a peer-reviewed literature survey using a series of keywords and combinations in Scopus. The following keywords were used: Biomass, case study(ies), clear-cut, close(r) to nature, combined objective, especially managed, Europe, even age(d), exotic plantation(s), forest management, forest(s), forestry, intensity, intensive, harvesting, logging, low intensity, native, natural, near-virgin forest(s), newly untouched, non-native, mixed-species, old-growth forest(s), plantation, planted, planting, primary forest(s), protective, recreational, regeneration, rotation, self-sown, short rotation, silviculture, timber, wood, uneven age(d), unmanaged forest(s), and untouched forest(s). Additionally, we used the name of European countries in the keywords combinations. The archetypes and the case studies were plotted in a matrix to assess the capacity of the archetypes to represent the case studies.

The resulting archetypes inherit the spatial units of assessment from the corresponding categories of naturalness and management, i.e. stand and landscape. Therefore, the stand level was adopted as the minimum unit of assessment for the archetypes, delivering therefore archetypes that are homogeneous in terms of both naturalness and management attributes. However, the landscape scale could additionally be considered in the archetypes resulting from the higher category of naturalness used in this study, i.e. near-virgin forests (see Table 1), which often represent large spatial units.

## Results

**Fig. 1:**
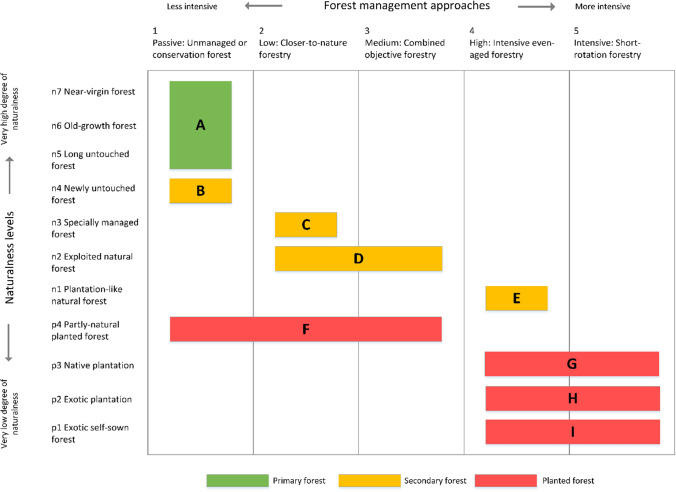
Archetype typology of forest ecosystems associating naturalness and forest management. Archetypes: **A** primary forests, **B** newly untouched forest, **C** specially managed forest under closer-to-nature forestry, **D** exploited natural forest under closer-to-nature or combined objective forestry, **E** plantation-like natural forest under intensive even-aged forestry, **F** partly natural forest under passive to medium intensity forest management, **G** native plantation under intensive even-aged or short-rotation forestry, **H** exotic plantation under intensive even-aged or short-rotation forestry, **I** exotic self-sown forest under intensive even-aged or short-rotation forestry

The resulting archetype typology delineates nine forest archetypes representative of European forest ecosystems (A to I in Fig. 1). The archetypes are organised from high to low degree of naturalness from top to bottom and from less to more intensive management from left to right. This results in a gradient where the archetypes are generally positioned along the main diagonal of the typology, describing qualitative relationships between naturalness and management intensity.

Next, we describe the archetypes in terms of naturalness, management, origin of the stand, structure, and biodiversity.

Archetype A—Primary forest: This archetype hosts the highest degree of naturalness and is characterised by three sub-types of primary forests, namely n7 Near-virgin forest, n6 Old-growth forest, and n5 Long untouched forest. These sub-types represent the last remnants of primary forest in Europe. They are relatively intact forests that exhibit no human impacts or have been without human impacts for at least sixty to eighty years. These forests are either unmanaged or managed solely for nature conservation purposes. Often, they are designated as nature reserves, where natural processes and disturbance regimes are allowed to develop without direct intervention.

Forests in late-seral stages in this archetype are characterised by high living biomass densities, accumulation of lying and standing deadwood, the presence of old trees, natural species composition and age structure, multiple canopy layers, and ecosystem functions. In addition, these forests feature canopy gaps and understory patchiness, both of which are key elements for natural regeneration, age structure, and rich biota. Forests in this archetype evolve according to natural disturbance regimes. Therefore, patches in early and mid-seral stages are also frequent in this archetype.

Archetype B—Newly untouched forest: This archetype describes forests with a moderately high degree of naturalness, although they are not considered primary forests. Forestry operations have either been discontinued or have never occurred since the establishment of the stand. Forests in this archetype are known to have been free from direct human disturbances for less than sixty to eighty years. However, signs of former silvicultural activities are generally evident. As a result, such stands may exhibit some similarities to archetype A in terms of composition, structure, and functions, albeit with a lower degree of naturalness. If the discontinuation of forestry operations is due to long rotation periods, the stand should be categorised as belonging to subsequent archetypes that have lower naturalness characteristics.

Archetype C—Specially managed forest under closer-to-nature forestry: Forests in this archetype exhibit a medium degree of naturalness and some significant old-growth attributes. The long continuity of low-intensity management and natural regeneration has given rise to naturalness traits such as structural diversity and often rich biodiversity.

The aim of closer-to-nature forestry is to manage forests by emulating natural processes. Therefore, natural-like species composition, an uneven-age structure, moderate to very high levels of deadwood, and natural regeneration are common characteristics of forests in this archetype.

Harvesting operations are designed to preserve the ecological functions of the forest, as well as to protect abiotic elements, such as the soil and watercourses, along with other natural habitats within the forest and their buffer zones. Clear-cutting is not allowed, with selective harvesting being the generally the preferred option.

Archetype D—Exploited natural forest under closer-to-nature or combined objective forestry: This archetype describes forests with a medium to low degree of naturalness that have been modified by silvicultural operations in such a way that the forest structure and species composition may be significantly different from the original natural state. However, self-sown native trees are generally present in forests of this archetype. This archetype also includes exploitations that modify old-growth forests.

Forests in this archetype are subject to medium to low management intensity, corresponding to combined objective forestry or to closer-to-nature forestry, respectively. In these cases, multiple management objectives can be met. These objectives may include, aside from wood production, soil and water protection, prevention of natural hazards (e.g. fire, avalanches, landslides), nature protection and conservation, and recreation.

The method of regeneration is generally natural regeneration, and tree species mixtures are typical for the forest type in question. However, planting or seedling are also acceptable methods for the (re)introduction of native species. Stands are generally mixed and uneven-aged. Harvesting operations are limited to stemwood, which favours the presence of deadwood on the forest floor and the maintenance of other characteristics of natural forests.

Archetype E—Plantation-like natural forest under intensive even-aged forestry: This archetype represents forests with a very low degree of naturalness. It specifically includes forests of self-sown native trees that are subjected to high-intensity management. This management approach creates forests that are similar to plantation forests, characterised by an even-aged structure, relative low age, regular spacing between trees, and the presence of only one or two tree species.

This archetype describes intensive even-aged forestry with the primary management objective of wood production for material or energy use. Natural regeneration is generally the preferred method within this archetype, although planting, coppicing, and seeding are also viable options. When planting is employed, the material can be sourced from tree breeding facilities. Forests in this archetype typically consist of a single tree species or occasionally two species if that enhances wood production. Pre-commercial thinning is carried out, and the final harvest method is usually clear-cutting. Alternatively, a combination of clear-cutting and shelterwood management systems may be used if it reduces the costs of establishment.

Archetype F—Partly natural forest under passive to medium intensity forest management: This archetype describes forests with a low degree of naturalness, composed of planted or sown native trees. However, the structure exhibits a higher degree of naturalness by being uneven-aged with mixed species or having a significant proportion of self-sown trees.

Stands in this archetype can result from different pathways. For instance, (i) the stand was not intensively managed for wood production after planting, (ii) the stand shows a large proportion of ageing trees and is subject low-intensity management, or (iii) the stand has remained unmanaged after planting. Therefore, the degree of naturalness in this archetype is not univocally associated with a specific forest management approach. On the contrary, it may correspond to forests that are currently unmanaged or abandoned, as well as those under closer-to-nature management or forestry with combined objectives. When planting is employed, the material can be sourced from tree breeding facilities.

Archetype G—Native plantation under intensive even-aged or short-rotation forestry: This archetype, characterised by an extremely low degree of naturalness, represents even-aged monocultures predominantly consisting of native tree species. The stands are stablished artificially through planting or sowing with regular spacing. The planting material can be sourced from breeding facilities. The main objective of the intensive even-aged forestry approach, which generally correspond to forests in this archetype, is wood production. Pre-commercial thinning is carried out in the forests represented by this archetype. Occasionally, this archetype may also be associated with intensive short-rotation forestry.

Archetype H—Exotic plantation under intensive even-aged or short-rotation forestry: This archetype, possessing almost no degree of naturalness, describes exotic plantations of fast growing species, where non-native trees are planted or sowed in regular arrays to create even-aged stands. The planting material, often produced via genetic modification, is sourced from breeding facilities. The primary objective of intensive even-aged forestry and short rotation forestry is production of wood for industrial processes or energy. Pre-commercial thinning is carried out, and the final harvesting system generally involves clear-cutting. In the case of short rotation, this approach is combined with the removal of all woody residues.

Archetype I—Exotic self-sown forests under intensive even-aged or short-rotation forestry: This archetype, which exhibits a variable degree of low naturalness, encompasses exotic forests that have grown from self-sown trees. Pre-commercial thinning is carried out in the forests represented by this archetype. In some cases, forests within this archetype can spread undesirably at the landscape level, such as the instance of invasive species. This archetype shares similarities with archetype H. For example, if there is uncertainty about whether a stand is self-sown, it may be classified as archetype H.

### Archetypes verification using case studies

The literature review yielded references describing case studies of forests, showcasing a spectrum of naturalness and management approaches across Europe. Out of approximately 300 references retrieved, 31 provided sufficient information for profile 38 case studies. From the 31 references, we extracted information about the case studies, covering general aspects (such as location and forest type), as well as more specific details, including the history of the forest sites (e.g. whether they originated from planted or sown forest, or natural regeneration), past forest use (e.g. whether they were managed or unmanaged), current forest management and silvicultural practices, age structure, tree species composition, and other characteristics relevant to matching the case studies with the characteristics of naturalness and forest management. Thus, we assigned each case study to a specific degree of naturalness and management approach using the information in Tables 1, 2, S1, and S2. Table S5 presents the references and the corresponding case studies, along with their designated level of naturalness and forest management approach.

The results of the comparison between the archetypes and case studies indicate that the archetype typology accurately encompasses the diversity of forest ecosystems across Europe (Table S6). Each of the 38 case studies corresponds to one of the nine archetypes, and no case study was left without a corresponding archetype. Some archetypes, such as, for example, archetype F, are broader, representing three distinct management approaches, as evidenced by seven case studies. In contrast, other archetypes are more specific; for instance, archetype E corresponds to one management approach and one level of naturalness, as seen in one case study. In summary, the archetype typology has proven to be an effective conceptual tool for describing the various associations between naturalness and management in European forests.

## Discussion

While the relationship between forest management and naturalness has been frequently addressed (Liira and Sepp 2009; Duncker et al. 2012a; Winter 2012; Messier et al. 2022), to our knowledge, no conceptual archetype typology existed. The results of this study help fill this gap. The archetype typology provides a synthetic framework that simplifies the understanding the complex links between forest management intensity and naturalness. It helps to clarify how human modification and forest use impact the degree of naturalness. The typology also aids in understanding the long-term effects of varying management approaches on forest ecosystems, particularly regarding those traits that result from the interplay between human action and nature. Changes in forest structural, compositional, and genetic traits can be driven by different degrees of forest management intensity. This, in turn, influences the functional traits of forest ecosystems, as well as the biological diversity and resilience of the ecosystem. The archetype typology confirms that higher levels of management intensity generally yield forest ecosystems with lower levels of naturalness (e.g. Barrette et al. 2020; Myllymäki et al. 2023), which are associated with reduced functions, services, and biodiversity (Cardinale et al. 2012; Gamfeldt et al. 2013; Watson et al. 2018). This outcome has significant implications for forest restoration and plans to increase forest resilience.

The use of planting material from breeding facilities, which in some cases includes material produced through genetic modification, may offer short-term benefits for wood production in planted forests (Ruotsalainen 2014). However, replacing the genetic profiles of native local species with seedlings from breeding facilities, which may correspond to plants from other regions even when the species is the same, can alter ecosystem resilience. For instance, the sustained use of uniform regeneration material or material from inappropriate genetic sources may result in forests with reduced genetic diversity and, hence, reduced resilience. Alterations of the genetic profiles may lead to potential genetic homogenisation and loss of intraspecific genetic diversity within the tree population (Olden and Rooney 2006), which is crucial for local adaptation and phenotypic plasticity. Both qualities are necessary for a species’ survival in the face of novel environmental stressors (Mackey et al. 2008; Watson et al. 2018). The genetic strategies for planted forests under rapid climate change must focus on maintaining species diversity and genetic diversity within species. For instance, this could be achieved by allocating areas for assisted regeneration to trees from regional provenances and from climate regimes that approximate projected climatic conditions (Thompson et al. 2009). Although resilience is influenced by various levels of biodiversity organisation, the genetic traits of species are considered the most important (Thompson et al. 2009). In summary, resilience emerges from the interplay of gene and species diversity, functional groups of species at multiple scales, and processes operating within the ecosystem (Gunderson 2000; Drever et al. 2006).

The verification of the archetype typology through 38 case studies significantly demonstrates its usefulness by utilising ground truth information. Analysis of the case studies reveals that the archetypes accurately capture the diversity of forest management approaches, forest history, and levels of naturalness in a sample across European forest ecosystems. The archetype typology could serve as a valuable tool for forest managers, conservation biologists, and policymakers in formulating strategies aimed at enhancing ecosystem resilience and preserving biodiversity. It is well-documented that forests closer their natural state tend to be more resilient than those modified by human activities (Loreau et al. 2001; Franklin et al. 2002; Seidl et al. 2014; Scherrer et al. 2023). With this in mind, initiatives to restore forest ecosystems and protect biodiversity can be seen as complementary to efforts to increasing resilience (Kuuluvainen and Aakala 2011; Winter 2012). A fact that is especially pertinent in a continent where the majority of forest land is available for wood supply. The formidable challenge posed by the uncertainties associated with anthropogenic climate change should be addressed through forest diversification, recognising that no single approach will be suitable for all situations, including options for management strategies (Thompson et al. 2009). In particular, planted forest (archetypes E, F, G, H, and I) could benefit from increased tree species diversity and in situ genetic diversity, programmes aimed at diversifying maladapted, low-diversity stands, and improved functional landscape connectivity, among other strategies (see: Messier et al. 2022). In terms of the archetype typology, this would involve, for instance, shifting the proportion of stands from low naturalness archetypes to those with a higher level of naturalness, at landscape level. The temporal and spatial dimensions of forest ecosystem restoration should be taken into account because enhancing resilience is a process that should be measured, relative to changes in forest traits, in terms of years to decades or hundreds of years, and spatially from patches to stands and up to entire landscapes.

Some limitations of this study stem from the frameworks adopted for forest management and naturalness. Although both management intensity and naturalness represent continuums (Winter et al. 2010; Duncker et al. 2012a), the classification frameworks adopted here are useful approaches for systematically understanding the full range of naturalness and management intensity occurring in European forest ecosystems. We acknowledge that any classification system inherently contains a degree of arbitrariness necessary for category separation. Furthermore, some categories may not be mutually exclusive, as is the case with the forest management categories used in this study (Duncker et al. 2012a). This introduces an inherent flexibility in the archetypes, encompassing everything from primary forests to fully anthropogenic forests, such as plantations. Consequently, some archetypes represent more than one category of either naturalness or management intensity (Fig. 1).

While maps depicting forest naturalness are scarcely available for large regions (Chiarucci and Piovesan 2020), maps describing forest management intensity have been created for the European continent (Hengeveld et al. 2012; Nabuurs et al. 2019). These maps can be instrumental in accounting for the area of each management category, and the corresponding naturalness, at different spatial scales, using the archetype typology. This option, which warrants further research, can provide baseline information for guidance on restoration needs, pursuing the aim of increased forest resilience. That is, managing forest in a way that approximates a more natural condition, thus exhibiting a higher level of naturalness.

## Supplementary Information

Below is the link to the electronic supplementary material.

Supplementary file 1 (PDF 854 KB)
