# Supplementary material for: Archetypal typology of European forest ecosystems integrating management intensity and naturalness
Source: Ambio. 2024 Jul 11;53(11):1587–98. doi: 10.1007/s13280-024-02050-3 (PMC11436663; doi:10.1007/s13280-024-02050-3)
Supplement: Supplementary file 1 — (PDF 854 KB) [file 13280_2024_2050_MOESM1_ESM.pdf]

Ambio

Supplementary Information - Appendix
This supplementary information has not been peer reviewed.

Title: **Archetypal typology of European forest ecosystems integrating management intensity and naturalness**

Table S1. Forest naturalness levels. Categories n8 to n10 not shown because they are very marginal in Europe. These categories were considered to be covered by category n7. Source: Modified from Buchwald (2005).

**Characteristics of forest naturalness levels - Summary**
n7 Near-virgin forest<br>- Untouched long enough to have developed structures, dynamics and species composition similar to virgin forest, even though they may have been significantly modified by human action in the past.<br>- Mixture in time and space between different seral stages, e.g. between old-growth stages and younger stages.<br>- Human impact is not obvious.
n6 High degree of naturalness - Old-growth forest<br>- Distinguished by old trees and related structural attributes.<br>- Old growth encompasses the later stages of stand development.<br>- Old-growth is distinguished from younger growth by several of the following attributes: 1) large trees for species and site, 2) wide variation in tree sizes and spacing, 3) accumulations of large-size dead standing and fallen trees that are high relative to earlier stages, 4) decadence in the form of broken or deformed tops or bole and root decay, 5) multiple canopy layers, and 6) canopy gaps and understory patchiness.<br>- Old-growth can develop following human disturbances.
n5 Quite high degree of naturalness - Long untouched forest<br>- Relatively intact forest unmodified by human activity for the past sixty to eighty years or for a relatively long time.<br>- Signs of former human impacts may still be visible, but strongly blurred due to the decades without forestry operations.
n4 Moderately high degree of naturalness – Newly untouched forest<br>- Forests where forestry operations have been discontinued or never occurred since stand establishment, and which are known to have been left untouched for less than sixty to eighty years.<br>- Signs of former management are usually easily visible.<br>- In principle any stand would belong here between forestry operations. If discontinuation of operations is only because of long management intervals, the stand is referred to lower levels.
n3 Medium degree of naturalness – Specially managed forest<br>- Forests with low-intensity use (presence of significant old-growth attributes).<br>- Biodiversity and often of cultural value. Examples are coppice, pasture forest, non-industrial selective logging and various stands of low accessibility or with protective or recreational functions.
n2 Fairly low degree of naturalness –Exploited natural forest<br>- Forests used and modified by humans so that the forest structure and species composition is more or less heavily changed from the originally natural situation, but still predominantly consisting of self-sown native trees, and without a plantation-like structure.<br>- Can be exploitation without preceding silviculture or forests with medium-intensity forest management.
n1 Low degree of naturalness – Plantation-like natural forest<br>- Forests predominantly consisting of self-sown native trees with high-intensity forest management.<br>- Forest structure has become plantation-like by being even-aged, having relatively low tree ages, fairly regular tree spacing and only one or two tree species in the canopy layer.
p4 Low degree of naturalness – Partly-natural planted forest<br>- Forests predominantly consisting of planted or sown native trees, and having somewhat natural structure by being un-evenaged, with mixed species or having significant ingrowth of self-sown trees, e.g. because multiple species have been planted and not intensively managed for timber or because of old age and low intensity of forestry.
p3 Extremely low degree of naturalness – Native plantation<br>- Intensively managed, even-aged forest predominantly consisting of native trees, established artificially by planting or sowing with regular spacing.<br>- Often monocultures, but sometimes two or more species are established together.
p2 Almost no degree of naturalness – Exotic plantation<br>- Forests predominantly consisting of non-native tree species.<br>- Stand origin is artificial by planting or sowing.
p1 Variably low degree of naturalness – Exotic self-sown forest<br>- Forests predominantly consisting of self-sown non-native tree species.<br>- In certain cases this category can spread at an undesirable scale, e.g. to the extent that it has replaced or seriously suppressed the species previously occupying the specific area.

Table S2. Forest management approaches. Source: modified from Duncker et al. (2012), and from European Commission (2023) for closer-to-nature forestry.

**Characteristics of forest management approaches – Summary**
1. Unmanaged or conservation forests<br>- The objective is to maintain ecologically valuable habitats and biodiversity.<br>- Natural processes and natural disturbance regimes develop without management intervention.<br>- No operations are allowed in forest reserves that might change the nature of the area.<br>- Stands have a history of development without direct management or exploitation.
2. Low — Closer-to-Nature Forestry<br>- The objective is to manage a forest with the emulation of natural processes as a guiding principle.<br>- Management approach closely resembling natural processes.<br>- Economic return is important but must occur within the frame of the principles of the approach.<br>- Management interventions enhance or conserve the ecological functions of the forest.<br>- Timber can be harvested and extracted during these activities, but most standing and fallen dead wood remains in the forest.<br>- Only native or site-adapted tree species are used.<br>- Method of regeneration is natural regeneration.<br>- Planting can be used to re-introduce native species, but genetically improved planting material cannot be used.<br>- Species mixtures follow the typical composition for the forest type.<br>- The final harvesting system should simulate natural disturbance mechanisms.<br>- Clear-cuts are not allowed.<br>- Extraction of biomass limited to removal of the stems.<br>- Machine operations are limited to a minimum, with an emphasis on the protection of natural structures during silvicultural activities.
3. Medium — Combined Objective Forestry<br>- In this approach various management objectives can be combined for satisfying diverse needs better than through zoning, where individual objectives are maximized in separate areas.<br>- Economic and ecological aims play a major role in this approach.<br>- In addition to timber production, other objectives may include habitat, water, and soil protection, mushroom production, game management, nature protection, avalanche and fire prevention, and recreation.<br>- There is great variability of silvicultural practices in this approach.<br>- Native or introduced tree species suitable for the site can be used.<br>- The preferred method of regeneration is natural regeneration, but planting or seeding is acceptable to introduce native or exogenous species.<br>- Planting material can be derived from tree breeding facilities, but genetically modified planting material is not used.<br>- Tree species mixtures are typical for the forest type.<br>- Site cultivation and fertilization are possible.<br>- Chemical pest control can be used in major outbreaks, which are either introduced from the surrounding stands or place the latter at risk.<br>- Minor outbreaks should not be treated with pesticides, and natural measures are preferred for pest control as well as to increase resilience (for example, greater use of mixed species stands).<br>- The rotation length is often longer than the age of maximum mean annual volume increment provided that financial criteria do not dictate otherwise.<br>- Biological legacies and natural habitats are often promoted inside the stands.<br>- Pre-commercial thinning can be carried out for reducing the number of trees.<br>- The final harvesting system is compatible with the chosen regeneration method.<br>- The intensity of harvesting is generally limited to solid wood volume, i.e. stems and branches.<br>- Vehicle movement is restricted to a strip road system.
4 High — Intensive Even-Aged Forestry<br>- Main objective of this approach is wood production.<br>- Characterised by forests in which no or relatively small age differences occur among individual trees.<br>- Typical stands consist of even-aged monocultures.<br>- Any non-invasive tree species suitable for the region can be chosen.<br>- Planting material can be derived from tree breeding facilities, but genetically modified planting material should not be used.<br>- Planting, coppice, seeding, and natural regeneration are all possible regeneration methods.<br>- Monocultures with small percentages of mixed-species stands are used in this approach (economic objective).<br>- Site preparation is often used to enhance establishment success, and remedial fertilization is used to increase growth rates.

- The rotation length depends mainly on economic returns.<br>- Pre-commercial thinning is carried out.<br>- Biomass extraction is commonly limited to solid wood volume, but might include whole-tree extraction, e.g. for bioenergy.<br>- Machine operations are not limited.<br>- The final harvest system is clear-cut or a combination of shelterwood and clear-cut if natural regeneration is preferred to reduce the costs of establishment.
5 Intensive—Short-Rotation Forestry<br>- The main objective is to produce the highest amount of timber or wood biomass.<br>- Economic objectives are given priority. Ecological concerns play a minor role in this approach.<br>- Selection of tree species depends mainly on economic return.<br>- Planting material can be derived from tree breeding facilities or produced via genetic modification.<br>- No natural colonization by other tree species is permitted if it reduces the growth of the chosen tree species.<br>- Sites are mechanically cultivated and can also be drained or irrigated if needed.<br>- Fertilization and liming are applied to enhance growth.<br>- The rotation length only depends on the economic return, often 20 years or less, and no biological legacies are included.<br>- No other habitats are maintained.<br>- Pre-commercial thinning is carried out.<br>- Intensity of machine operations is maximum compared with the other approaches.<br>- The final harvesting system is a clear-cut combined with removal of all woody residues.

Table S3. Indicators and descriptors of forest naturalness and management approaches. Columns n7 to p1 and FMA 1 to FMA 5 show the descriptors assigned to each naturalness level and management approach, respectively. For a description of the naturalness levels see Table S1, and for the management approaches Table S2.

		Naturalness levels											Management approaches				
**Indicators**	**Descriptors**	**n7**	**n6**	**n5**	**n4**	**n3**	**n2**	**n1**	**p4**	**p3**	**p2**	**p1**	**FMA 1**	**FMA 2**	**FMA 3**	**FMA 4**	**FMA 5**
**Structural**																	
Age structure	1: Uneven; 2: Even	1	1	1	1	1	1	2	1	2	2	1	1	1	1	2	2
Deadwood	1: High; 2: Moderate; 3: Low	1	1	1	1, 2	1	2, 3	3	2, 3	3	3	3	1	1, 2	2, 3	3	3
Veteran/large trees (presence)	1: Yes; 2: No	1, 2	1	1, 2	1, 2	1	1, 2	2	1, 2	2	2	2	1, 2	1, 2	2	2	2
Canopy layers	1: Single; 2: Multiple	2	2	2	2	2	2	1	2	1	1	2	2	2	1, 2	1	1
Seral stages	1: Yes; 2: Temporarily; 3: No	1	1	1	1	1	1, 2	3	1, 2	3	3	3	1	1	2	3	3
Late seral stage communities	1: Present; 2: Not present	1, 2	1	1, 2	2	1	1, 2	2	2	2	2	2	1, 2	1, 2	2	2	2
Variation in tree size and spacing	1: High; 2: Low; 3: Absent	1	1	1	1	1	1	3	1	3	3	2	1	1	1, 2	3	3
Canopy gaps and understory patchiness	1: Yes; 2: No	1	1	1	1	1	1, 2	2	1	2	2	1, 2	1	1	2	2	2
Micro habitats	1: Yes; 2: No	1	1	1	1, 2	1	1, 2	2	1	2	2	2	1	1, 2	2	2	2
**Functional**																	
Regeneration	1: Natural; 2: Planting / seedling; 3: Coppice	1	1	1	1	1	1	1	2	2	2, 3	1	1	1	1, 2	2	2, 3
**Compositional**																	
Tree species diversity	1: Mixed; 2: Mono-specific	1	1	1	1	1	1	1,2	1	2	2	2	1	1	1	1, 2	2
Tree species composition	1: Natural (PNV); 2: Human-modified	1	1	1	1	1, 2	1, 2	2	1, 2	2	2	2	1	1, 2	2	2	2
Tree species origin	1: Native; 2: Introduced	1	1	1	1	1	1	1	1	1	2	2	1	1	1, 2	1, 2	2
**Human impact**																	
Signs of management	1: Yes; 2: No	2	2	1, 2	1	1	1	1	1	1	1	1	2	1	1	1	1
Signs of harvesting	1: Evident; 2: Blurred; 3: Not evident	2, 3	2, 3	2, 3	1, 2	1	1	1	1, 2, 3	1	1	1, 3	2, 3	1	1	1	1
Use intensity (harvesting)	1: No use; 2: Low; 3: Medium; 4: High	1	1	1	1	2	3	4	1, 2	4	4	4	1	2	3	4	4
Protective/recreational functions	1: Possible; 2: No	1	1	1	1	1	1	2	1, 2	2	2	2	1	1	1	2	2

Chemical inputs	1: Possible; 2: No	2	2	2	2	2	2	1, 2	2	1	1	1	2	2	1	1	1
Maturity (rotation)	1: No intervention; 2: Long; 3: Medium; 4: Short; 5: Shortest	1	1	1	1	2	2, 3	4	1, 2, 3	4, 5	4, 5	4, 5	1	2	3	4	5

Table S4. Hamming distance calculated between naturalness levels and forest management approaches. The lower the Hamming distance, the more similar the categories are. The forest archetypes delineated are in light grey. For a description of the naturalness levels and management approaches see Table S1 and Table S2, respectively.

		**Forest management approach**				
		**FMA 1**	**FMA 2**	**FMA 3**	**FMA 4**	**FMA 5**
**Naturalness level**	**n7**	0	0.21	0.53	0.79	0.89
	**n6**	0	0.21	0.63	0.89	1
	**n5**	0	0.16	0.47	0.74	0.84
	**n4**	0.05	0.11	0.32	0.63	0.74
	**n3**	0.21	0	0.47	0.74	0.84
	**n2**	0.26	0.05	0.05	0.47	0.58
	**n1**	0.68	0.47	0.32	0.05	0.16
	**p4**	0.16	0.05	0.21	0.47	0.58
	**p3**	0.84	0.63	0.37	0	0.05
	**p2**	0.89	0.68	0.37	0	0
	**p1**	0.63	0.47	0.26	0.21	0.21

Table S5. Case studies and references collected in the literature review along with the assigned management approach and level of naturalness.

**Case study (reference)**	**Forest management approach (FMA) (Duncker et al. 2012)**	**Naturalness level (Buchwald 2005)**	**Description**
1: Forest nature reserve in Białowieża National Park, Poland (Duncker et al. 2012)	1	n7	Primary forest nature reserve in Białowieża National Park, Poland.
2: European beech (*Fagus sylvatica* L.) forests in Baden-Wuerttemberg, Germany (Duncker et al. 2012)	2	n2	The forest type is European beech (*Fagus sylvatica* L.) forest with coniferous admixture. Beech forests represent the natural forest in this region. The long term forest development objective is semi-natural, well structured European beech stands with significant admixtures of conifers. The regeneration method is natural regeneration. If there is insufficient natural regeneration, beech is planted with additional planting of site adapted mixed species. Generally only solid wood is removed. The production cycle is in the range of 80 to 150 years. The final felling system is mostly selective cutting. This case study can be summarised as a low intensity management with some medium intensity measures.
3: Norway spruce (*Picea abies* L.) forests in Västerbotten county in Sweden (Duncker et al. 2012)	3	p4	Management in this case study refers to the forest type "mixed forests dominated by Norway spruce (*Picea abies* L.)". The main objective is wood production. The preferred method of regeneration is planting after clear-cut or natural regeneration with a shelterwood system. The final harvest system is preferably clear-cut.
4: Sitka spruce (*Picea sitchensis*) forests in Scotland (Duncker et al. 2012)	4	p2	Fast growing plantation forests. The balance between timber production, conservation, recreation and amenity depends on local conditions. Stands managed so that pulpwood and small roundwood is produced in early thinnings while saw timber is provided by later thinning and final clear-cutting.
5: *Eucalyptus* stands (Duncker et al. 2012)	5	p2	*Eucalyptus* stands in Portugal.
6: Carpathian beech (*Fagus*) forests (Kulla et al. 2023)	1	n6	European beech (*Fagus sylvatica* L.) old-growth forests in the Western Carpathians, Central Europe. Strictly protected nature reserves.
7: Gribskov forest in Denmark (Meinhard Hallund 2019)	2	p4	Gribskov is a productive forest with natural, cultural and recreational values. A transition from an intensive management to close-to-nature management took place in 2005. Parts of these forests are designated as a Natura 2000 site. This case study represents forests in transition in which legacies from previous management systems will be present for a considerable time.

			Therefore, despite being under close-to-nature forestry it was allocated to a low naturalness category according to the information provided in the source.
8: Exotic plantation in Arigna Region (Ireland) (Fléchard et al. 2006)	4	p2	In this case study afforestation started in the late 1960's and culminated at the end of the 1990's. Conifers represented most of the total forest cover in the area in 2003 and it is estimated that planted Sitka spruce (*Picea sitchensis*) represents about 80% of this coniferous cover.
9: Forest in Lahemaa and Karula National Parks (Estonia) (Köster et al. 2005)	1	n4	Conifer-dominated forest landscapes in Lahemaa and Karula National Parks. Stands with no records of silvicultural activity since the 1920s.
10: Lowland forests in Northern Italy (Agestam et al. 2018)	2	p4	This case study describes 350 ha of lowland forests, which are 60-70-year-old planted forests (pine species), semi-natural lowland forest remnants, and some 20 year old afforested and reforested areas. Part of the area in included in the Natura 2000 network. The area presents limited wood production. However, it is important for tourism, recreation activities, and non-wood products. Forest management operations are not primarily aimed to wood production, and are oriented to more close-to-nature conditions.
11: Unmanaged ancient forests in the Southern French Alps (Marage and Lemperiere 2005)	1	n4	Unmanaged Silver fir (*Abies alba*) ancient forests of the southern French Alps. Forests corresponding to potential natural vegetation and unexploited for more than half a century.
12: Abernethy forest in northern Scotland (Mason et al. 2007)	1	p4	Abernethy forest within the Cairngorms National Park is considered to form part of the native pinewoods of Scotland and have been dominated by Scots pine (*Pinus sylvestris* L.) for several millennia. From ~1780 until 1850, there were intesivelly harvested resulting in severe depletion. A change of ownership in the 1980s resulted in a greater emphasis on conservation. The area shows no evidence of timber harvesting for the last 30 years and is considered to be developing towards typical natural structure pinewoods.
13: Multiple purpose forest management. The Queen Elizabeth Forest in Scotland (Kazana et al. 2003)	3	n2	The Queen Elizabeth Forest covers 67 000 hectares and includes areas of native woodland and productive forests. The area is managed to deliver multiple objectives, including timber production, biodiversity conservation, water supply and recreation.
14: Veluwe forest (the Netherlands) (Yousefpour et al. 2015)	3	p4	Forests composed by a mosaic of different stands managed for different purposes under a multifunctional schema. The management objectives are regeneration to a more natural state, recreation and wood production. The forest area is dominated by Scots pine (*Pinus sylvestris* L.) that was planted in the 19th and 20th centuries. Followed by semi-natural mixed forest.

15: Conversion of plantations of exotic coniferous species into more natural forests (Drenthe, The Netherlands). (Jonášová et al. 2006)	1	p4	Case study describing management oriented to conversion of plantations of exotic coniferous species into more natural forests in The Netherlands. The main management objective in this region is to increase the proportion of indigenous broadleaved trees and to convert pure coniferous stands into mixed stands. Artificial gaps in the plantations were created and followed by natural regeneration of both indigenous and exotic species.
16: Forest in the Swiss Plateau (Blattert et al. 2018)	3	n2	In this case study we consider the management approach and forest type until 2007 because after that year the forest area was not managed for timber production anymore. The forest is structured in mixed even-aged stands. The general silvicultural system is group selection with the use of harvesting machines. Clear-cutting was not allowed for areas greater than 0.5 ha owing to local regulations. A mix of tree species was used in the stands.
17: Short-rotation forestry for biomass production in Flanders (Belgium) (Vande Walle et al. 2007)	5	p2	Plantations established on former agricultural land by hand planting at Zwijnaarde. The plantations are composed of birch (*Betula pendula* Roth), maple (*Acer pseudoplatanus* L.), poplar (*Populus trichocarpa×deltoides*) and willow (*Salix viminalis*). The plantation activity was preceded by tillage of the upper 20 cm of the soil. The only management objectives is biomass production.
18: Pinus plantation in the Basque Country (Spain) (Gartzia-Bengoetxea et al. 2009)	4	p2	The study case represents even-aged *Pinus radiata* D. plantations in northern Spain. Logging operations include forest harvesting with skidders and site preparation including ploughing. During site preparation the residues of the previous plantation are removed.
19: Pratomagno forest (Italy) (De Meo et al. 2020)	3	n2	The Pratomagno forest area covers 3000 ha. The dominant species are European beech (*Fagus sylvatica* L.) and Turkey oak (*Quercus cerris* L.), while black pine (*Pinus nigra* spp.) stands resulting from reforestation cover 800 ha. The management is oriented to a transition toward mixed forests characteristic of the area dominated by European beech, chestnut (*Castanea sativa*), and oak (*Quercus* L.) species. Thinning operations are conducted regularly.
20: Old growth forest in the High Coast Region in Sweden (Sandström et al. 2020)	1	n6	Scots pine (*Pinus sylvestris* L.) forests in the High Coast Area representative of undisturbed forests with a high degree of naturalness and exhibiting old-growth characteristics.
21: Late-successional *Picea abies* L. northern Fennoscandia (Finland) (Aakala et al. 2009)	1	n7	Late-successional *Picea abies* L. stands in northern boreal zone in Fennoscandia. Pallas-Yllästunturi National Park in north-western Finland.
22: Late-successional *Picea abies* L. northern Fennoscandia (Russia) (Aakala et al. 2009)	1	n7	Late-successional *Picea abies* L. stands in northern boreal zone in Fennoscandia. Kazkim River area in Murmansk province, north-western Russia.

23: Old-growth forest in Vallone Cervara forest (Italy) (Burrascano et al. 2008)	1	n6	Protected old growth forest European beech (*Fagus sylvatica* L.) stand in the Vallone Cervara forest.
24: European beech (*Fagus sylvatica* L.) managed as shelterwood (Burrascano et al. 2008)	3	n2	European beech (*Fagus sylvatica* L.) managed as a shelterwood, located in Vallone Ciafassa, last harvested in 1976. Stand characterised by even-aged structure and one-layered canopy.
25: Augsburg Western Forest and Lieberose-Schaubetal-Neuzelle forest (Germany) (Agestam et al. 2018)	3	n2	This case study represent two state-owned forests. The Augsburg Western Forest (AWF) in the federal state of Bavaria, and the Lieberose-Schaubetal-Neuzelle (LSN) forest in the federal state of Brandenburg. In the AWF the dominant species are Norway spruce (*Picea abies* L.) (62% of standing volume) and beech (*Fagus*) (11%). The most common silvicultural practices are shelterwood, clear-cutting or selection systems with Norway spruce, and selection or non-uniform shelterwood system with beech. In the LSN Scots pine (*Pinus sylvestris* L.) (65% of standing volume) and oak (11%) are the most important species. The most frequent silvicultural practices are uniform shelterwood systems and clear-cutting with Scots pine, and selection or shelterwood systems with oak.<br>Both areas are dominated by a mix of practices oriented to multifunctional forest management.
26: Forest in Wild Nephin National Park in County Mayo (Ireland) (Agestam et al. 2018)	1	n4	Wild Nephin is a wilderness area where exotic fast growing lodgepole pine (*Pinus contorta*) stands were commercially thinned and was then left to develop into a natural parkland, undergoing a rewilding programme to allow the bog and forest go back to their natural state. Wild Nephin National Park covers a total of 15 000 hectares of different ecosystems.<br>The national park includes 4000 hectares of commercial forests (Nephin Forest) taken over in 2017. Since then reforestation using native species including sessile oak (*Quercus petraea*), birch (*Betula* L.), rowan (*Sorbus*), alder (*Alnus*), poplar (*Populus* L.) and native Scots pine (*Pinus sylvestris* L.), have taken place in a model of “return to wilderness”.
27: *Eucalyptus* plantation in Serra de Monchique (Portugal) (Barrocas et al. 1998)	5	p2	Plantations of *Eucalyptus globulus* Labill. in Serra de Monchique. *Eucalyptus* has been extensively planted and exploited because of its fast growing character and profitability in short periods of time.
28: *Eucalyptus* plantation in northern Spain (Cabanillas Roldán 2019)	5	p2	Plantation areas primarily assigned to timber harvesting, mainly eucalyptus (*Eucalyptus globulus* Labill.) and less commonly pine (*Pinus*), chestnut (*Castanea*) or oak (*Quercus*).
29: *Cedrus deodara* plantation in northern Italy (Manzone 2016)	5	p2	*Cedrus deodara* is a fast growing tree species planted for woodchips production for energy use.

30: Uneven-aged forest in the Western Carpathians (Romania) (Banaś et al. 2018)	2	p4	Case study in the Szczawiczne forest, covering an area of 456 ha in the Western Carpathians. The forest is under uneven-aged forest management consistent with close-to-nature silviculture since the 1970s. The forest exhibits a wide variety of species composition and age structures at the landscape level. In the past, the forest was managed by the shelterwood system with mostly even-aged stands.
31: Native plantations in Sweden (Drössler et al. 2015)	4	p3	Case study established in 1994 in an afforestation landscape in southern Sweden with 66 stands, 18 of which planted with single tree species, including native Swedish tree species.
32: Exotic self-sown *eucalyptus* in Spain and Portugal (Calviño-Cancela and Rubido-Bará 2013; Catry et al. 2015; Fernandes et al. 2018)	Naturalised from FMA5	p1	*Eucalyptus* tree species self-sown and naturalised in Spain and Portugal.
33: Scots pine (*Pinus sylvestris* L.) plantations in Scotland (Mason et al. 2007)	4	p3	Even-aged Scots pine (*Pinus sylvestris* L.) plantations in Scotland. The prevailing silvicultural system is patch clear-felling with planting and a rotation length of 60–80 years.
34: Low intensity forestry in montane forests in the Italian Alps (Motta et al. 2015)	2	n3	Case study describing mixed beech (*Fagus sylvatica* L.), silver fir (*Abies alba*), and Norway spruce (*Picea abies* L.) uneven-aged montane forests in Santo Stefano di Cadore, Croviana and Amblar, all above the 1000 m.a.s.l. in the Italian Alps. These forests exhibit continuous canopy cover and multilayered vertical structure. Silvicultural practices are summarised in harvesting of single tree to small group of trees, maintaining a multi-layered and uneven-aged stands, with limited impact on the landscape. Selection cuttings are repeated every 8 to 10 years, and the main goal of the management is the conservation of the desired diameter distribution and species composition.
35: Naturally regenerated maritime pine (*Pinus pinaster* Aiton) in Southern Europe (Ribeiro et al. 2022)	4	n1	Case study describing natural regenerated stands of native maritime pine (*Pinus pinaster* Aiton) in Portugal, Spain (norther plateau), and France (southwestern region, notably on coastal areas and Aquitaine). Silvicultural practices focus on different harvesting systems including clear-cutting, cutting in progressive strips, shelterwood system, and clear-cutting in patches, with harvesting intensities from moderate to high.
36: Forest in the Dürrenstein Wilderness Area in Austria (Albrich et al. 2021)	1	n5	The Dürrenstein Wilderness Area is a protected area located in eastern Austria covering an extension of 3449 ha. The area includes formerly managed forests which were subject to selective cutting of conifers, and more recently clear-cut systems with rotation periods of between 100 and 160 years. The dominant tree species are European beech (*Fagus sylvatica* L.), Norway spruce (*Picea abies* L.) and silver fir (*Abies alba*). The area representing this case

			study has been without management for at least 100 years, and have developed several old-growthness features.
37. Chestnut (*Castanea sativa* Mill.) stands in Monte Amiata, Italy (Manetti et al. 2022)	5	p3	Case study describing stands of native chestnut (Castanea sativa Mill.) coppices for wood production in Monte Amiata in Central Italy. The area extend over 3534 ha. In most of the area (87%) the silvicultural system consist of coppices with short rotation periods (<20 years), no thinning, and a high number of standards (up to 60–80 per hectare).
38. Douglas-fir (*Pseudotsuga menziesii*) stands in Eastern Austria and Southern Germany (Eberhard et al. 2021)	4	p1	This case study represents 434 Douglas-fir (*Pseudotsuga menziesii*) stands managed by private forest companies in Eastern Austria and Southern Germany. Douglas-fir is an exotic tree species original from North America. Natural regeneration under the shelter of mature trees by opening up or group removal is one of the approaches used by the forest managers in these areas.

Table S6. Comparison of forest ecosystems archetypes A to I, in grey, and case studies 1 to 38 described in Table S5.

	**Forest management approach (intensity)**					
**Naturalness level**	FMA 1: Passive—Unmanaged or conservation forests	FMA 2: Low—Closer-to-Nature Forestry	FMA 3: Medium —Combined Objective Forestry	FMA 4: High—Intensive Even-Aged Forestry	FMA 5: Intensive—Short-Rotation Forestry	**Total**
n7–Near-virgin forest	**A:** 1, 21, 22					**3**
n6–Old-growth forest	6, 20, 23					**3**
n5–Long-untouched forest	36					**1**
n4–Newly-untouched forest	**B**: 9, 11, 26					**3**
n3–Specially managed forest		**C**: 34				**1**
n2–Exploited natural forest		**D**: 2	13, 19, 25, 24, 16			**6**
n1–Plantation-like natural forest				**E**: 35		**1**
p4–Partly-natural planted forest	**F**: 12, 15	7, 10, 30	3, 14			**7**
p3–Native plantation				**G**: 31, 33	37	**3**
p2–Exotic plantation				**H**: 4, 8, 18	5, 17, 27, 28, 29	**8**
p1–Self-sown exotic forest				**I**: 38	32	**2**
**Total**	**12**	**5**	**7**	**7**	**7**	**38**

References Appendix

Aakala T., T. Kuuluvainen, T. Wallenius, and H. Kauhanen. 2009. Contrasting patterns of tree mortality in late-successional Picea abies stands in two areas in northern Fennoscandia. *Journal of Vegetation Science* 20: 1016-1026. https://doi.org/10.1111/j.1654-1103.2009.01100.x

Agestam E., K. Wallertz, and U. Nilsson 2018. Alternative Forest Management Models for ten Case Study Areas in Europe. ALTERFOR project WP1, Deliverable 1.2, 72 p.

Albrich K., D. Thom, W. Rammer, and R. Seidl. 2021. The long way back: Development of Central European mountain forests towards old-growth conditions after cessation of management. *Journal of Vegetation Science* 32: e13052. https://doi.org/10.1111/jvs.13052

Banaś J., S. Zięba, and L. Bujoczek. 2018. An Example of Uneven-Aged Forest Management for Sustainable Timber Harvesting. *Sustainability* 10: 3305. https://doi.org/10.3390/su10093305

Barrocas H.M., M.M. da Gama, J.P. Sousa, and C.S. Ferreira. 1998. Impact of reafforestation with Eucalyptus globulus Labill. on the edaphic collembolan fauna of Serra de Monchique (Algarve, Portugal). *Miscellania Zoologica* 21: 9-23.

Blattert C., R. Lemm, O. Thees, J. Hansen, M.J. Lexer, and M. Hanewinkel. 2018. Segregated versus integrated biodiversity conservation: Value-based ecosystem service assessment under varying forest management strategies in a Swiss case study. *Ecological Indicators* 95: 751-764. https://doi.org/10.1016/j.ecolind.2018.08.016

Buchwald E., 2005. A hierarchical terminology for more or less natural forests in relation to sustainable management and biodiversity conservation, Third expert meeting on harmonizing forest-related definitions for use by various stakeholders. Food and Agriculture Organization of the United Nations, Rome, pp. 111-127.

Burrascano S., F. Lombardi, and M. Marchetti. 2008. Old-growth forest structure and deadwood: Are they indicators of plant species composition? A case study from central Italy. *Plant Biosystems - An International Journal Dealing with all Aspects of Plant Biology* 142: 313-323. https://doi.org/10.1080/11263500802150613

Cabanillas Roldán D. 2019. Centipede biodiversity (Myriapoda, Chilopoda) in two eucalyptus plantations in northern Spain and other records from Vegadeo (Asturias, Spain). *Boletín de la Asociación Española de Entomología* 43: 287-303.

Calviño-Cancela M., and M. Rubido-Bará. 2013. Invasive potential of Eucalyptus globulus: Seed dispersal, seedling recruitment and survival in habitats surrounding plantations. *Forest Ecology and Management* 305: 129-137. https://doi.org/10.1016/j.foreco.2013.05.037

Catry F.X., F. Moreira, E. Deus, J.S. Silva, and A. Águas. 2015. Assessing the extent and the environmental drivers of Eucalyptus globulus wildling establishment in Portugal: results from a countrywide survey. *Biological Invasions* 17: 3163-3181. https://doi.org/10.1007/s10530-015-0943-y

De Meo I., P. Cantiani, and A. Paletto. 2020. Effect of Thinning on Forest Scenic Beauty in a Black Pine Forest in Central Italy. *Forests* 11: 1295. https://doi.org/10.3390/f11121295

Drössler L., R. Övergaard, P.M. Ekö, P. Gemmel, and H. Böhlenius. 2015. Early development of pure and mixed tree species plantations in Snogeholm, southern Sweden. *Scandinavian Journal of Forest Research* 30: 304-316. https://doi.org/10.1080/02827581.2015.1005127

Duncker P.S., S.M. Barreiro, G.M. Hengeveld, T. Lind, W.L. Mason, S. Ambrozy, and H. Spiecker. 2012. Classification of Forest Management Approaches: A New Conceptual Framework and Its Applicability to European Forestry. *Ecology and Society* 17. https://doi.org/10.5751/es-05262-170451

Eberhard B.R., T. Eckhart, and H. Hasenauer. 2021. Evaluating Strategies for the Management of Douglas-Fir in Central Europe. *Forests* 12: 1040. https://doi.org/10.3390/f12081040

European Commission 2023. Guidelines on Closer-to-Nature Forest Management. European Commission, Luxembourg, 98 p. https://doi.org/10.2779/731018

Fernandes P., C. Máguas, O. Correia, and P. González-Moreno. 2018. What drives Eucalyptus globulus natural establishment outside plantations? The relative importance of climate, plantation and site characteristics. *Biological Invasions* 20: 1129-1146. https://doi.org/10.1007/s10530-017-1614-y

Fléchard M.-C., Á.N. Dhubháin, M. Carroll, and P. Cohn, 2006. Forestry and the local community in Ireland: A Case Study in the Arigna Region, Small-scale Forestry and Rural Development: The intersection of ecosystems, economics and society, Galway, Ireland, pp. 81-92.

Gartzia-Bengoetxea N., A. González-Arias, E. Kandeler, and I.M.d. Arano. 2009. Potential indicators of soil quality in temperate forest ecosystems: a case study in the Basque Country. *Annals of Forest Science* 66: 303-303. https://doi.org/10.1051/forest/2009008

Jonášová M., A. van Hees, and K. Prach. 2006. Rehabilitation of monotonous exotic coniferous plantations: A case study of spontaneous establishment of different tree species. *Ecological Engineering* 28: 141-148. https://doi.org/10.1016/j.ecoleng.2006.05.008

Kazana V., R.H. Fawcett, and W.E.S. Mutch. 2003. A decision support modelling framework for multiple use forest management: The Queen Elizabeth Forest case study in Scotland. *European Journal of Operational Research* 148: 102-115. https://doi.org/10.1016/S0377-2217(02)00348-X

Köster K., K. Jõgiste, H. Tukia, M. Niklasson, and T. Möls. 2005. Variation and ecological characteristics of coarse woody debris in Lahemaa and Karula National Parks, Estonia. *Scandinavian Journal of Forest Research* 20: 102-111. https://doi.org/10.1080/14004080510042137

Kulla L., J. Roessiger, M. Bošeľa, S. Kucbel, V. Murgaš, J. Vencurik, J. Pittner, P. Jaloviar, et al. 2023. Changing patterns of natural dynamics in old-growth European beech (Fagus sylvatica L.) forests can inspire forest management in Central Europe. *Forest Ecology and Management* 529: 120633. https://doi.org/10.1016/j.foreco.2022.120633

Manetti M.C., M. Conedera, F. Pelleri, P. Montini, A. Maltoni, B. Mariotti, M. Pividori, and E. Marcolin. 2022. Optimizing quality wood production in chestnut (Castanea sativa Mill.) coppices. *Forest Ecology and Management* 523: 120490. https://doi.org/10.1016/j.foreco.2022.120490

Manzone M. 2016. Quality, productivity, energy and costs of woodchip produced by Cedrus deodara plantations: A case study in Italy. *Biomass and Bioenergy* 92: 81-87. https://doi.org/10.1016/j.biombioe.2016.06.008

Marage D., and G. Lemperiere. 2005. The management of snags: A comparison in managed and unmanaged ancient forests of the Southern French Alps. *Annals of Forest Science* 62: 135-142. https://doi.org/10.1051/forest:2005005

Mason W.L., T. Connolly, A. Pommerening, and C. Edwards. 2007. Spatial structure of semi-natural and plantation stands of Scots pine (Pinus sylvestris L.) in northern Scotland. *Forestry: An International Journal of Forest Research* 80: 567-586. https://doi.org/10.1093/forestry/cpm038

Meinhard Hallund A., 2019. Close-to-nature forest management in Gribskov - An evaluation of land use distribution and biomass occurrence, Department of Geosciences and Natural Resource Management. University of Copenhagen.

Motta R., M. Garbarino, R. Berretti, F. Meloni, A. Nosenzo, and G. Vacchiano. 2015. Development of old-growth characteristics in uneven-aged forests of the Italian Alps. *European Journal of Forest Research* 134: 19-31. https://doi.org/10.1007/s10342-014-0830-6

Ribeiro S., A. Cerveira, P. Soares, and T. Fonseca. 2022. Natural Regeneration of Maritime Pine: A Review of the Influencing Factors and Proposals for Management. *Forests* 13: 386. https://doi.org/10.3390/f13030386

Sandström J., M. Edman, and B.G. Jonsson. 2020. Rocky pine forests in the High Coast Region in Sweden: structure, dynamics and history. *Nature Conservation* 38. https://doi.org/10.3897/natureconservation.38.34870

Vande Walle I., N. Van Camp, L. Van de Casteele, K. Verheyen, and R. Lemeur. 2007. Short-rotation forestry of birch, maple, poplar and willow in Flanders (Belgium) I—Biomass production after 4 years of tree growth. *Biomass and Bioenergy* 31: 267-275. https://doi.org/10.1016/j.biombioe.2007.01.019

Yousefpour R., M. Didion, J.B. Jacobsen, H. Meilby, G.M. Hengeveld, M.-J. Schelhaas, and B.J. Thorsen. 2015. Modelling of adaptation to climate change and decision-makers behaviours for the Veluwe forest area in the Netherlands. *Forest Policy and Economics* 54: 1-10. https://doi.org/j.forpol.2015.02.002
